# MicroRNAs serve as prediction and treatment-response biomarkers of attention-deficit/hyperactivity disorder and promote the differentiation of neuronal cells by repressing the apoptosis pathway

**DOI:** 10.1038/s41398-022-01832-1

**Published:** 2022-02-19

**Authors:** Liang-Jen Wang, Ho-Chang Kuo, Sheng-Yu Lee, Lien-Hung Huang, Yuyu Lin, Pei-Hsien Lin, Sung-Chou Li

**Affiliations:** 1 Department of Child and Adolescent Psychiatry, Kaohsiung Chang Gung Memorial Hospital and Chang Gung University College of Medicine, Kaohsiung, Taiwan; 2 Department of Pediatrics, Kaohsiung Chang Gung Memorial Hospital and Chang Gung University College of Medicine, Kaohsiung, Taiwan; 3 Kawasaki Disease Center, Kaohsiung Chang Gung Memorial Hospital, Kaohsiung, Taiwan; 4 Department of Psychiatry, Kaohsiung Veterans General Hospital, Kaohsiung, Taiwan; 5 Department of Psychiatry, College of Medicine, Graduate Institute of Medicine, School of Medicine, Kaohsiung Medical University, Kaohsiung, Taiwan; 6 Center for Mitochondrial Research and Medicine and Genomics and Proteomics Core Laboratory, Department of Medical Research, Kaohsiung Chang Gung Memorial Hospital and Chang Gung University College of Medicine, Kaohsiung, Taiwan

**Keywords:** Clinical genetics, Diagnostic markers

## Abstract

Attention-deficit/hyperactivity disorder (ADHD) is a highly heritable neurodevelopmental disorder. This study aimed to examine whether miRNA expression abundance in total white blood cells (WBCs) facilitated the identification of ADHD and reflected its response to treatment. Furthermore, whether miRNA markers facilitated the growth of the human cortical neuronal (HCN-2) cells was also investigated. Total WBC samples were collected from 145 patients and 83 controls, followed by RNA extraction and qPCR assays. Subsequently, WBC samples were also collected at the endpoint from ADHD patients who had undergone 12 months of methylphenidate treatment. The determined ΔCt values of 12 miRNAs were applied to develop an ADHD prediction model and to estimate the correlation with treatment response. The prediction model applying the ΔCt values of 12 examined miRNAs (using machine learning algorithm) demonstrated good validity in discriminating ADHD patients from controls (sensitivity: 96%; specificity: 94.2%). Among the 92 ADHD patients completing the 12-month follow-up, miR-140-3p, miR-27a-3p, miR-486-5p, and miR-151-5p showed differential trends of ΔCt values between treatment responders and non-responders. In addition, the in vitro cell model revealed that miR-140-3p and miR-126-5p promoted the differentiation of HCN-2 cells by enhancing the length of neurons and the number of junctions. Microarray and flow cytometry assays confirmed that this promotion was achieved by repressing apoptosis and/or necrosis. The findings of this study suggest that the expression levels of miRNAs have the potential to serve as both diagnostic and therapeutic biomarkers for ADHD. The possible biological mechanisms of these biomarker miRNAs in ADHD pathophysiology were also clarified.

## Introduction

Attention-deficit/hyperactivity disorder (ADHD) is a neurodevelopmental disorder characterized by inattention, hyperactivity, and/or impulsivity [1]. The prevalence rate of ADHD among school-age children is as high as 3–10% [2]. Recent studies have yielded estimates between 70 and 80% for the heritability of ADHD, and complex epigenetic factors play an important role in the etiology of this condition [3]. MicroRNAs (miRNAs) are small noncoding RNAs that downregulate gene expression in human cells [4]. miRNAs function in central nervous system development, such as cell proliferation and differentiation, synaptogenesis, synaptic plasticity, and apoptosis. Therefore, miRNAs might be the key to identify novel biomarkers for the diagnosis and/or prognosis of ADHD [5–7]. Researchers have demonstrated the role of gene polymorphisms within miRNA target sites in ADHD pathogenesis among human subjects [8, 9]. Several case-control studies have revealed that miRNAs can potentially serve as biomarkers to differentiate ADHD patients from healthy subjects [10–14]. Some studies have used next-generation sequencing (NGS) technology to explore ADHD-related miRNAs with unknown biological mechanisms [15, 16]. Our research team previously also applied NGS to identify WBC miRNAs as high-performance biomarkers for identifying ADHD [17].

Pharmacotherapy, particularly psychostimulant medication, remains a treatment option for ADHD [18]. Methylphenidate (MPH), the most widely used and therapeutically efficient drug for ADHD, exerts its pharmacological effects by increasing the levels of dopamine and norepinephrine in the synaptic cleft [19]. Recognition and management of ADHD in children is important so that their long-term outcomes can be improved [20, 21]. Few studies have investigated the relationship between miRNA expression in blood and ADHD prognosis. A previous study indicated that the expression level of miRNA let-7d significantly changed after a 6-week treatment with repetitive transcranial magnetic stimulation (rTMS) and atomoxetine in ADHD patients [22]. Another study demonstrated that during 3- and 6-month follow-up, clinical attention deficit symptoms were negatively correlated with the relative expression of miR-4655-3p and miR-7641 [23]. Some available data have suggested that therapy with psychotropic drugs (specifically, antipsychotics, antidepressants, and mood stabilizers) appears to have complex and region-dependent effects on epigenetic mechanisms [24]. MPH has been shown to upregulate the expression of Homer 1a and to ameliorate ADHD-like behaviors [25]. To date, however, no study has investigated the longitudinal trend of miRNA expression in ADHD patients under MPH treatment.

For the mechanisms underlying the association between ADHD and miRNAs, animal studies have investigated whether miRNA (i.e., rno-let-7d) expression or miRNA target genes (i.e., *Homer 1a*) are related to an ADHD phenotype [25–29]. A rat model revealed that miR-1-b, miR-182, miR-183-5p, miR-206-3p, miR-211-5p, miR-384-5p, miR-471-5p, and miR-741-3p may be involved in learning and memory deficits in ADHD [30, 31]. Dopamine deficiency has been proposed as an underlying cause of ADHD, and upregulation of miR-140-5p and miR-140-3p was observed in dopamine neurons [32]. A dual-luciferase reporter assay further indicated that hsa-miR-3171 binding might alter SLC1A3 gene expression [33]. A bioinformatics analysis indicated that single nucleotide polymorphisms (SNPs) within miRNA recognition elements of the genes might confer susceptibility to ADHD [8]. Our previous study using structural magnetic resonance imaging (MRI) indicated that the gray matter (GM) volume was negatively correlated with the ΔCt values of miR-126-5p, miR-140-3p, and miR-30e-5p [34]. However, the mechanisms by which these miRNAs modulate neuronal growth and influence GM volume remain unclear.

Therefore, this study aimed to examine whether our miRNA diagnostic panel established using NGS and qPCR [17] could differentiate ADHD patients from healthy subjects in an independent cohort. Second, this study aimed to discover whether miRNAs can function as biomarkers that reflect the condition of ADHD under treatment. Third, using a human neuronal cell line (HCN-2) in an in vitro study, we aimed to determine whether three miRNAs (miR-126-5p, miR-140-3p, and miR-30e-5p) associated with GM volume affected the growth of the HCN-2 cells.

## Materials and methods

### Study participants

The Institutional Review Board (IRB) at Chang Gung Memorial Hospital in Taiwan approved the protocol of this study. We obtained written informed consent from the parents or guardians of all patients and controls, in accordance with the Declaration of Helsinki.

In total, 145 eligible participants, comprising ADHD patients treated in the outpatient Department of Child Psychiatry at Chang Gung Memorial Hospital in Taiwan and healthy control children, were enrolled. The criteria for ADHD patients were as follows: (a) a clinical diagnosis of ADHD based on the criteria provided in the Diagnostic and Statistical Manual of Mental Disorders (DSM-5) [35, 36]; (b) age between 6 and 16 years; (c) drug-naïve status; and (d) Han Chinese ethnic background. The exclusion criteria included a history of major physical illness (such as genetic, neurological, metabolic disorders, or infectious conditions) or comorbid major neuropsychiatric diseases (such as autism spectrum disorder, intellectual disabilities, mood disorders, psychotic disorders, or severe brain injury).

Eighty-three children without ADHD were recruited as healthy control subjects within the same catchment area. Healthy controls were between the ages of 6 and 16 years and were ethnically Han Chinese. These participants were required to be children without any of the aforementioned physical illnesses or neuropsychiatric diseases.

### Study procedures and clinical measurements

A senior psychiatrist interviewed all the participants in both the ADHD patients and the control subjects using the DSM-5 and the ADHD Rating Scale (ADHD-RS) [37]. Subsequently, the Wechsler Intelligence Scale for Children–Fourth Edition (WISC-IV) [38] was administrated to individual patients by an experienced child psychologist in a room designed to reduce variability in testing conditions. Total WBC samples were collected in the outpatient department from all participants to test miRNAs levels.

Patients with ADHD underwent a 12 months of methylphenidate (MPH) treatment and follow-up. After completing testing at baseline (M0), the patients and their parents or caregivers were counseled regarding ADHD and drug therapy. During the follow-up period, the patients were prescribed a dose of MPH based on the severity of their clinical symptoms and their age, height, and body weight. The MPH formulations (IR-MPH or OROS-MPH) or dosages may have been modified for certain patients during the 12-month follow-up period. Drug compliance was confirmed at each visit based on reports by the patients’ parents or caregivers and the presentation of the remaining MPH medication.

The posttest (M12) occurred 12 months after baseline and the procedures performed at M0 were repeated. Total WBC samples were collected from participants again to test miRNA levels. Patients’ psychiatrists subsequently completed the ADHD-RS. Of the 145 patients, 92 patients completed the assessment at M12. Response to therapy was defined as a 30% improvement in symptoms compared to the ADHD-RS scores at baseline. In addition, the total ADHD-RS scores ≤ 18 [39, 40]. Of the 92 patients, 50 (54.3%) met the criteria for remission (responder group), while 42 (45.7%) did not achieve remission during the follow-up period (nonresponder group).

### Total WBC enrichment, RNA collection, and qPCR assays

We collected total WBCs, extracted RNA samples and conducted qPCR assays by referring to our previous study [17]. In summary, 5 mL of whole blood was collected from each subject with Vacutainer^®^ Blood Collection Tubes (with EDTA, REF367835, BD, New Jersey, USA), followed by RBC lysis. Then, the RBC-free pellets were collected to extract total RNA. Next, real-time quantitative reverse transcription-polymerase chain reaction (qRT-PCR) was applied to determine the expression profiles of 12 selected miRNAs (miR-140-3p, miR-27a-3p, miR-101-3p, miR-150-5p, let-7g-5p, miR-30e-5p, miR-223-3p, miR-142-5p, miR-486-5p, miR-151-3p, miR-151-5p, and miR-126-5p) with the small nucleolar RNA RNU44 as the endogenous control.

### HCN-2 cell culture and miRNA mimic transfection

Human cortical neuronal cells (HCN-2, CRL-10742, ATCC) were cultured in Dulbecco’s modified Eagle’s medium (DMEM, Gibco 11965092) supplemented with 4 mM L-glutamine (Gibco 25030149), 1.5 g/L sodium bicarbonate, 4.5 g/L glucose, and 10% fetal bovine serum (Gibco 10437028). miRNA mimics for hsa‐miR-126‐5p (YM00470487), hsa‐miR-140‐3p (YM00470393), and hsa‐miR-30e-5p (YM00470246), as well as scramble control (YM00479903) were obtained from QIAGEN. For miRNA mimic transfection, 2 × 10^5^ HCN-2 cells were first seeded on a 24‐well plate, and each well contained 500 μL of culture medium. After 24 h, 3 μL of miRNA mimic (20 μM) and 6 μL of HiPerFect transfection reagent (QIAGEN) were mixed in 200 μL of culture medium without fetal bovine serum (FBS). After 6 h, 600 μL of culture medium was added to the 24‐well plate, and the cells were cultured. Finally, 24 h after transfection, the cells were harvested for further assays.

### Recording and evaluating the differentiation patterns of HCN-2 cells

Twenty-four hours after transfection with miRNA mimic, the cells were observed to monitor cell differentiation. For each well, we recorded the differentiation patterns by taking three independent pictures at 100× magnification on days 1, 3, 6, and 9. As a result, for each treatment, 3 (wells) × 3 (replications) × 4 (time points) = 36 pictures were taken to evaluate the differentiation patterns. During the differentiation assay, we renewed the culture medium every 2 days. After the differentiation assays, the pictures were analyzed using AngioTool [41], a software designed to analyze the differentiation patterns of endothelial and neuronal cells.

### Microarray assays, qPCR assays and further data analyses

In addition to the differentiation assays, the cells were harvested to collect RNA samples 24 h after transfection. The RNA samples were subjected to qPCR assays to examine to what extent miRNA mimic transfection enhanced miRNA abundance levels. The collected RNA samples meeting the demand of having an RNA integrity number (RIN) ≥ 7 (RIN value determined using Agilent Bioanalyzer 2100) were also subjected to microarray assays. In summary, the RNA samples were prepared using WT-Plus kits (Thermo Fisher, Waltham, MA, USA), followed by hybridization on Human Clariom S microarray chips (Thermo Fisher, Waltham, MA, USA) and scanning using a Gene Scanner 3000 7 G (Thermo Fisher, Waltham, MA, USA). The generated cell files were analyzed using Partek to identify the differentially expressed genes, which were further applied to conduct pathway enrichment analysis. The microarray data have been submitted to NCBI GEO and is accessible via the accession number GSE189295.

### Flow cytometry

We conducted flow cytometry assays according to a previous study [42]. In summary, the transfected HCN-2 cells were stained using an Annexin V apoptosis detection kit (BD Pharmingen™, 556547). Detection of apoptosis by flow cytometry (BD LSR II) and classification of the four quadrants were performed as follows: the lower-left quadrant exhibits living cells (FITC−/PI−); the upper right quadrant indicates nonliving cells/late apoptotic cells (FITC+/PI+); the lower right quadrant demonstrates early apoptotic cells (FITC+/PI−); and the upper left quadrant suggests necrotic cells (FITC+/Pl−).

### Statistical analysis

We analyzed data using the statistical software package SPSS, version 20.0 (SPSS Inc., Chicago, IL, USA). The sample size was analyzed using the software package G-Power 3.1; based on the settings of 80% power, *p* = 0.05. Variables were presented as either the mean (standard deviation) or case numbers (frequency). Two-tailed *p* values < 0.05 were considered statistically significant. We applied the chi-square test to compare the sex distribution between the ADHD patients and the controls, and between ADHD treatment responders and nonresponders. The potential differences in continuous variables between groups were examined using independent t-tests.

The library for support vector machines (SVMs, LIBSVM) is integrated software for support vector classification, regression, and distribution estimation [43, 44]. The ΔCt values of selected miRNAs were composited to develop an ADHD prediction model with an SVM algorithm. Receiver operating characteristic (ROC) curves and the area under the curve (AUC) were utilized to evaluate both the specificity and sensitivity of the probability score yielded by the LIBSVM. The cutoff values at a probability score of 0.5 were regarded as the optimal diagnostic point of the signature.

A paired *t*-test was used to examine the potential differences in the ΔCt values of each miRNA during the one-year follow-up in responders and nonresponders. Furthermore, repeated-measures ANOVA was applied to examine the differential trends of the ΔCt values of miRNAs during the 1-year follow-up between responders and non-responders (patient age was included as a covariate). We used Pearson correlation to examine the potential relationships between the one-year change in ΔCt values of selected miRNAs and the 1-year change in ADHD-RS cores, in responders and non-responders.

## Results

### Demographic data

Table 1 summarizes the characteristics of the 145 ADHD patients and 83 healthy controls. Compared to the control group, the ADHD group had a higher proportion of males (76.6% male in the ADHD group and 56.6% male in the control group, *p* = 0.002) and was younger (mean age 8.9 years in the ADHD group and 9.9 years in the control group, *p* = 0.004). In addition, ADHD patients had a lower intelligence quotient (*p* < 0.001), higher inattention scores (*p* < 0.001), and higher hyperactivity/impulsivity scores (*p* < 0.001), as rated by parents or teachers (SNAP-IV) or clinicians (ADHD-RS).

**Table 1:** Characteristics of patients with ADHD and healthy controls.

Characteristics	ADHD (*N* = 145)	Controls (*N* = 83)	Statistic	*p* value
**Sex**			9.850	0.002*
Male	111 (76.6)	47 (56.6)		
Female	34 (23.4)	36 (43.4)		
**Age** (years)	8.9 ± 2.2	9.9 ± 2.6	2.901	0.004*
**WISC-IV**				
FSIQ	97.7 ± 10.7	107.8 ± 13.1	−6.243	<0.001*
Verbal Comprehension Index	101.0 ± 11.5	105.7 ± 11.8	−2.887	0.004
Perceptual Reasoning Index	98.5 ± 12.4	109.4 ± 15.9	−5.309	<0.001*
Working Memory Index	99.2 ± 12.8	107.4 ± 11.9	−4.750	<0.001*
Processing Speed Index	93.8 ± 9.3	101.5 ± 11.5	−5.456	<0.001*
**Clinical measures**				
SNAP-IV parent form (I)	16.5 ± 5.5	5.4 ± 5.8	14.288	<0.001*
SNAP-IV parent form (H)	14.5 ± 6.4	4.2 ± 5.3	12.928	<0.001*
SNAP-IV teacher form (I)	14.8 ± 5.8	4.2 ± 4.8	14.415	<0.001*
SNAP-IV teacher form (H)	11.9 ± 6.9	2.6 ± 3.4	13.109	<0.001*
ADHD-RS (I)	22.3 ± 4.6	1.2 ± 3.6	39.746	<0.001*
ADHD-RS (H)	23.6 ± 4.9	1.5 ± 4.3	35.433	<0.001*

^a^Data are expressed as N (%) or mean ± SD; *FSIQ* Full-Scale Intelligence Quotient, *H* hyperactivity/impulsivity scores, *I* inattention scores, *SNAP-IV* Swanson, Nolan, and Pelham Version IV Scale, *WISC-IV* Wechsler Intelligence Scale for Children–Fourth Edition; *p < 0.05.

### miRNAs served as ADHD biomarkers and enabled disease prediction

**Fig. 1:**
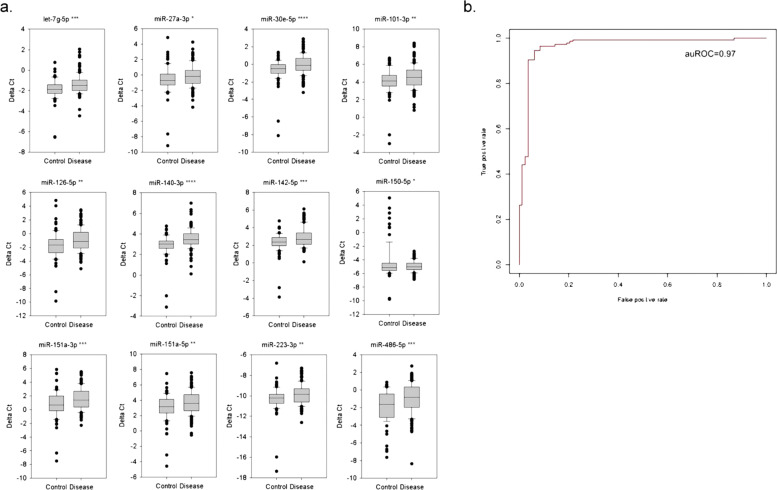
The miRNA expression profile and ADHD prediction model. **a** We evaluated 12 miRNA expression profiles in total WBCs from either healthy control (control, *N* = 83) subjects or ADHD patients (disease, *N* = 145). RNU44 was adopted as the internal control gene. **b** We used the delta Ct values of 12 miRNAs to develop an ADHD prediction model with an auROC value of 0.97. The *p* values of comparisons were derived with a *t*-test. *, **, *** and **** denote *p* value < 0.05, *p* value < 0.01, *p* value < 0.001, and *p* value < 0.0001, respectively.

We used qPCR assays to quantify the abundance of the 12 miRNAs in total WBCs from subjects. The qPCR kits and primers used were the same as those applied in our previous study [17]. After the qPCR assays, we acquired ΔCt values using *RNU44* as the internal control. As shown in Fig. 1a, all 12 miRNAs were significantly differentially expressed between the 145 ADHD (disease) and 83 healthy control (control) samples. This result was consistent with that of our previous study [17]. In addition, no miRNA was significantly altered when the samples were compared based on sex (158 males vs. 70 females) or age (the samples were divided into two sets based on age, median: 8.67 years).

We further used the ΔCt values of 12 miRNAs to develop an ADHD prediction model with a SVM algorithm. In summary, we first used a ten-fold policy to derive the optimal parameters for the sample set, namely, gamma = 0.0625 and cost = 128. Then, using the miRNA ΔCt values of 228 subjects as a training set (83 healthy controls vs. 145 ADHD subjects), we derived a high-performance prediction model with an ROC of 0.966 (Fig. 1b). Therefore, consistent with our previous study, miRNA profiles from total WBCs may serve as ADHD biomarkers, facilitating the prediction of ADHD.

### Changes in miRNA levels were associated with treatment response among ADHD patients

Of the 145 ADHD patients, 92 received MPH treatment and completed the 12-month follow-up. Supplementary Table 1 summarizes the characteristics of the 50 treatment responders (78% males) and 42 nonresponders (78.6% males). During the 1-year treatment period, the responders and nonresponders showed 55.1 ± 13.9% and 11.4 ± 15.4% decreases, respectively, in total ADHD-RS scores. Compared to the nonresponder group, the responder group was younger (mean age 8.2 years in responders and 9.3 years in non-responders, *p* = 0.008). The average daily doses of MPH in the responders and nonresponders were 27.0 ± 8.0 mg and 26.9 ± 7.9 mg, respectively.

**Fig. 2:**
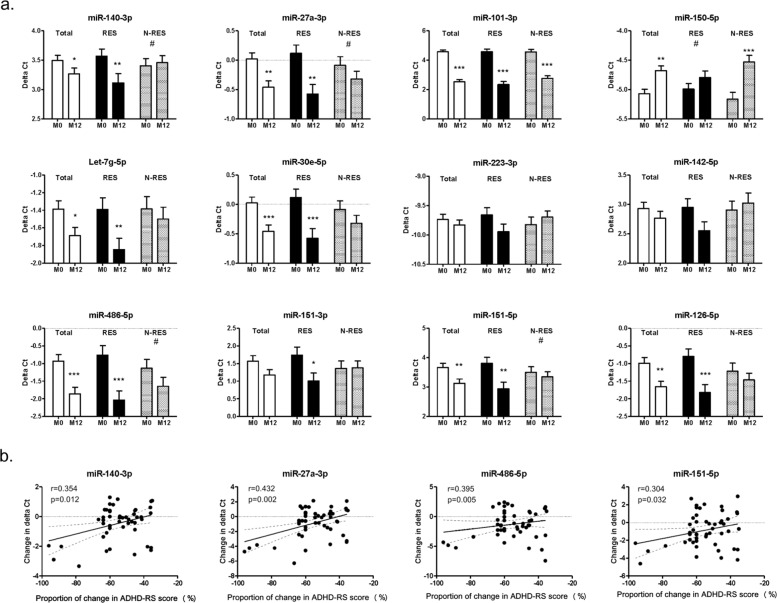
The miRNA expression profile in ADHD patients after 12-month methylphenidate treatment and their relationships with the proportion of change in total ADHD-RS scores. **a** The miRNA expression profile in total ADHD patients (*n* = 92), responders (RES, *n* = 50) and non-responders (N-RES, *n* = 42) after 12-month methylphenidate treatment. The *p* values of ΔCt values comparisons between baseline (M0) and the endpoint (M12) were derived with a paired *t*-test. *, **, *** and **** denote *p* value < 0.05, *p* value < 0.01, *p* value < 0.001, and *p* value < 0.0001, respectively. # denotes significant interaction effects of treatment response and time on ΔCt values (using repeated measure of ANOVA). **b** The relationships between changes in miRNAs and the proportion of change in total ADHD-RS scores among the responders during the 12-month follow-up. The changes in ADHD-RS scores showed a significantly positive correlation between changes in ΔCt values of miR-140-3p (*r* = 0.354, *p* = 0.012), miR-27a-3p (*r* = 0.432, *p* = 0.002), miR-486-5p (*r* = 0.395, *p* = 0.005), and miR-151-5p (*r* = 0.304, *p* = 0.032). The dashed lines indicate the 95% confidence interval of correlation.

In a total of 92 ADHD patients (Fig. 2a), the ΔCt values of eight miRNAs (miR-140-3p, miR-27a-3p, miR-101-3p, let-7g-5p, miR-30e-5p, miR-486-5p, miR-151-5p, and miR-126-5p) significantly decreased with MPH treatment for 12 months. However, the ΔCt value of miR-150-5p significantly increased. Further stratified analyses revealed that the ΔCt values of nine miRNAs (miR-140-3p, miR-27a-3p, miR-101-3p, let-7g-5p, miR-30e-5p, miR-486-5p, miR-151-3p, miR-151-5p, and miR-126-5p) in the responder group significantly decreased. In the non-responder group, the ΔCt values of miR-101-3p and miR-150-5p significantly decreased and increased, respectively.

### Relationships between the changes in miRNA abundances and clinical measures

After the 12-month follow-up, we further examined whether the changes in miRNA abundances (the variations in ΔCt values after 12 months) were correlated with the improved ADHD-RS scores. The comparison results were shown in Fig. 2b. Among the responders, the changes in ADHD-RS scores during the 12-month follow-up showed a significantly positive correlation with the changes in ΔCt values of miR-140-3p (*r* = 0.354, *p* = 0.012), miR-27a-3p (*r* = 0.432, *p* = 0.002), miR-486-5p (*r* = 0.395, *p* = 0.005), and miR-151-5p (*r* = 0.304, *p* = 0.032). Among the nonresponders, the changes in ADHD-RS scores did not show a significant correlation with the changes in the ΔCt values of any miRNAs during the 12-month follow-up. In addition, the daily doses of MPH in use were not significantly correlated with changes in ΔCt values of any miRNAs during the 12-month follow-up.

### miR-126-5p and miR-140-3p transfection promoted the differentiation of HCN-2 cells in vitro

**Fig. 3:**
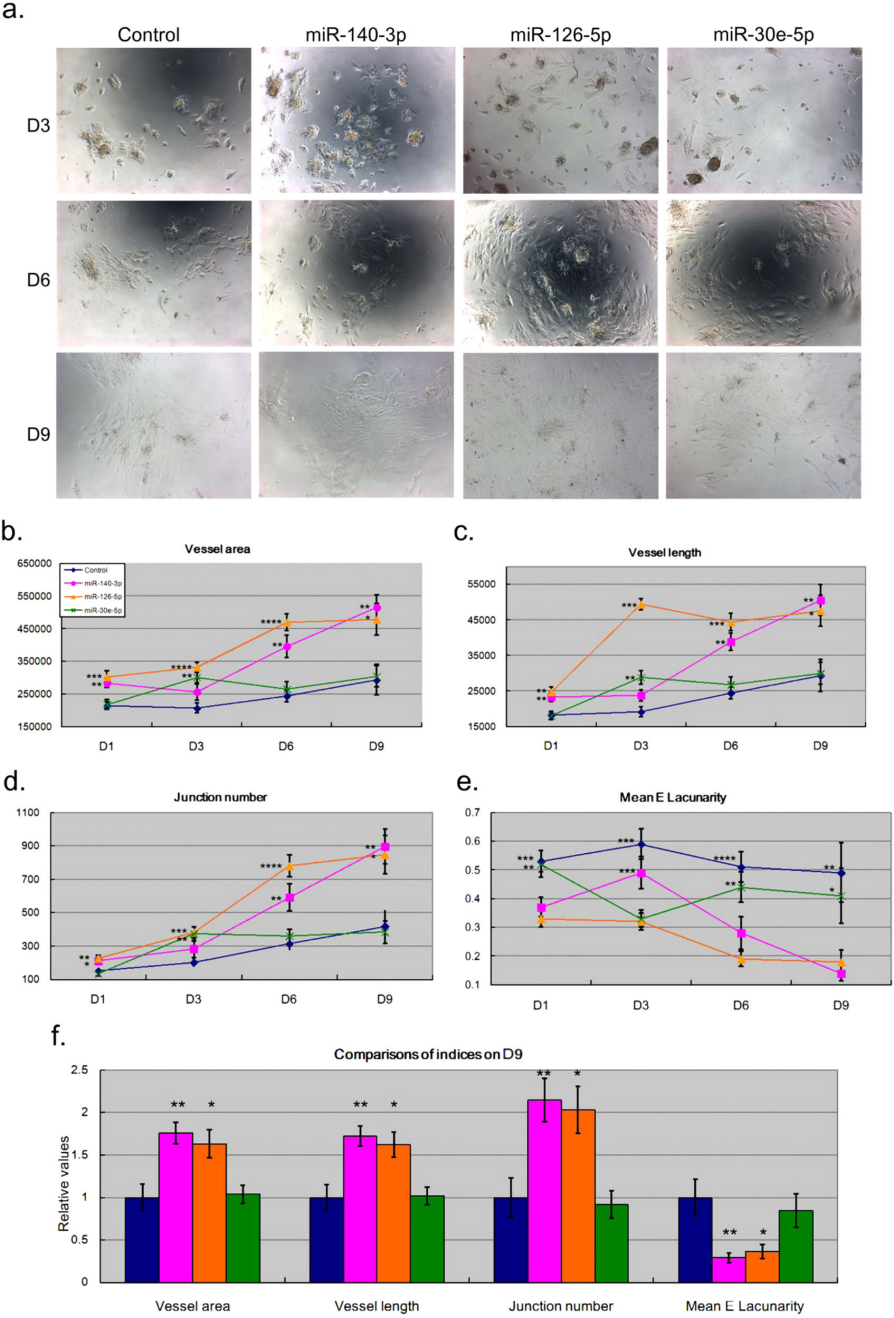
Illustration and comparisons of the differentiation patterns of HCN-2 cells transfected with miRNA mimic. **a** HCN-2 cells were transfected with scrambled control mimic (control), hsa-miR-30e-5p mimic (miR-30e), hsa-miR-140-3p mimic (miR-140), or hsa-miR-126-5p mimic (miR-126) for 24 h. Then, they were seeded on new plates for monitoring and recording growth with a camera. Each transfection treatment had three independent wells, and three pictures were randomly taken from each well, resulting in 3*3 = 9 pictures. (**b**–**f**) We determined the differentiation patterns of HCN-2 cells in terms of different indices, including (**b**) vessel area, (**c**) vessel length, (**d**) junction number, and (**e**) mean lacunarity. The *t*-test *p* values were calculated by comparing the values of the control set on a specific day. **f** For a systematic comparison, the values were normalized to the average values of the control set at day 9. *, **, *** and **** denote *p* value < 0.05, *p* value < 0.01, *p* value < 0.001, and *p* value < 0.0001, respectively.

To examine whether miRNAs affected the differentiation of neuronal cells, we transfected HCN-2 cells with miRNA mimics, followed by differentiation assays. As shown in Supplementary Fig. 1, miRNA mimic transfection enhanced the abundancesof miRNAs by approximately 600-fold or more, which reflected the success of miRNA mimic transfection in neuronal cells. Next, as shown in Fig. 3a, HCN-2 cells differentiated increasingly over time in all sets. On days 6 and 9, cells differentiated into tube structures rather than aggregating as cell masses on day 3. In addition, compared with the scrambled control, miR-126-5p and miR-140-3p seemed to better promote cell differentiation at days 6 and 9. For comprehensive and systematic comparisons, we used AngioTool [41] to analyze the pictures recording the differentiation patterns. As shown in Supplementary Fig. 2, AngioTool was designed to identify the tube structures (also called the vessel structures) by sketching the outline of vessels and recording their area. AngioTool also recorded the lengths of vessels with thick red lines. Moreover, the junctions within the vessel structures are also illustrated with blue dots.

AngioTool evaluates the differentiation of endothelial and neuronal cells by quantifying the vessel area, vessel length, junction number, and mean lacunarity. As shown in Fig. 3a, over time, the vessel area (Fig. 3b), vessel length (Fig. 3c), and junction number (Fig. 3d) gradually increased, and the mean lacunarity (Fig. 3e) gradually decreased. Such results were consistent with Fig. 3a and demonstrated that HCN-2 cells differentiated increasingly well overtime in each set. Moreover, since day 1, transfection with miR-140-3p and miR-126-5p resulted in significantly higher vessel area (Fig. 3a), vessel length (Fig. 3c), and junction number (Fig. 3d) and lower mean lacunarity (Fig. 3e) than transfection with the scrambled control set, demonstrating that both miR-140-3p and miR-126-5p promoted the differentiation of neuronal cells. For a systematic comparison, the values of indices at day 9 were normalized to one based on the control set (Fig. 3f). Consistent with previous subfigures, in addition to the promotion ability, miR-140-3p dominated over miR-126-5p in promoting the differentiation of neuronal cells.

### miR-126-5p and miR-140-3p promoted the differentiation of neuronal cells by repressing apoptosis

To understand the downstream pathways through which miR-140-3p and miR-126-5p promoted the differentiation of HCN-2 cells, we conducted microarray assays. Cells transfected with scrambled control miR-140-3p or miR-126-5p mimic were harvested for RNA extraction and microarray assays. As shown in Supplementary Fig. 3a, the three sets had markedly different individual gene expression profiles, which were clearly different from each other. Further analyses identified 2055 and 2159 differentially expressed genes (*p* < 0.05 and variation > 1.25-fold) in the miR-140-3p vs. control and miR-126-5p vs. control comparisons, respectively. We conducted cluster analysis on the 3700 genes formed by the union of these two sets of genes. As shown in Supplementary Fig. 3b, despite having their own individual gene expression profiles, the cells transfected with miR-140-3p or miR-126-5p mimic were clustered together and apart from the control set. This result was consistent with the idea that these two manipulations led to similar effects of promoting differentiation.

**Fig. 4:**
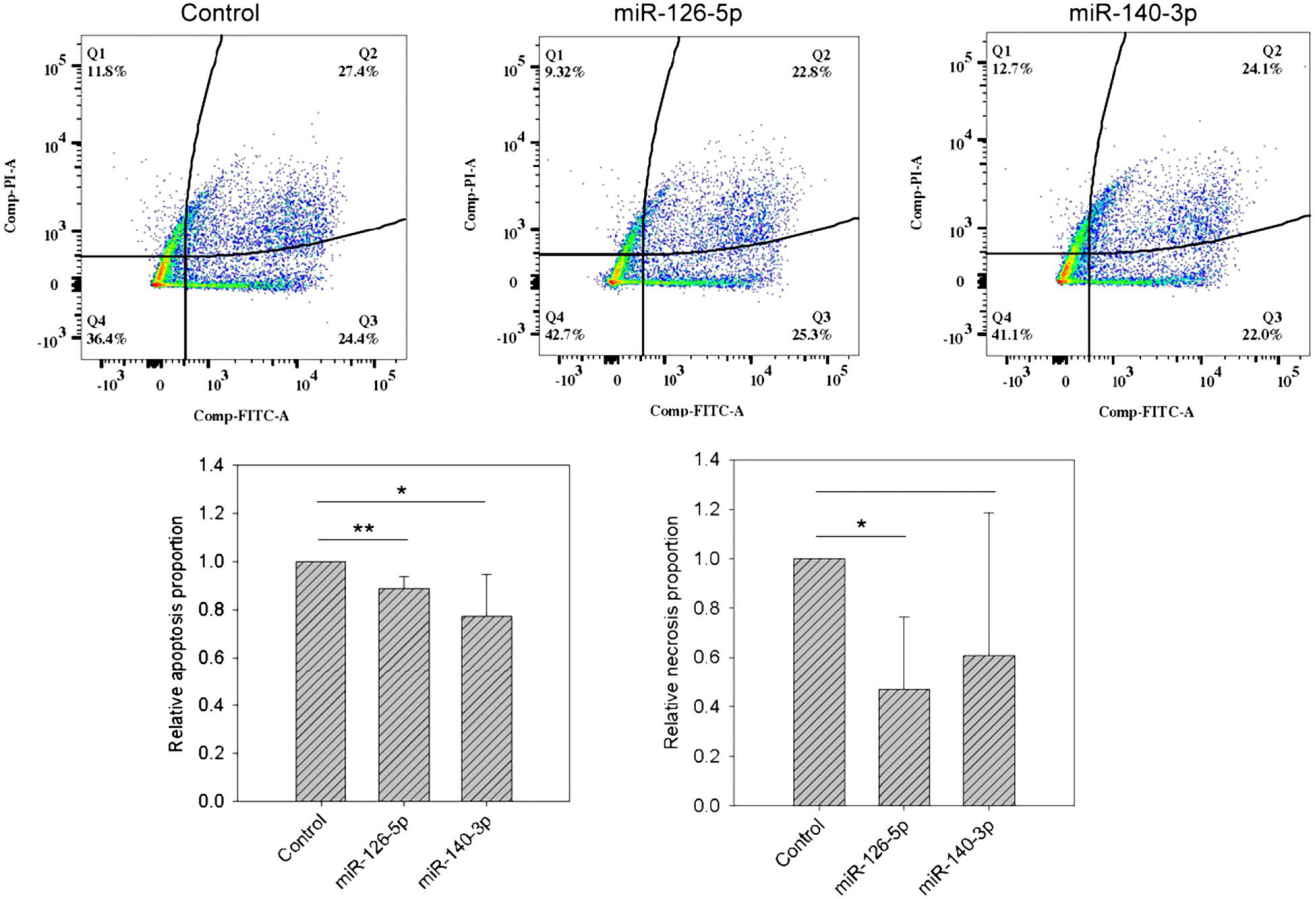
Flow cytometry assay results. We used a flow cytometry assay to determine the proportion of cells undergoing apoptosis and necrosis. The upper subfigures denote the results of one flow cytometry run. Through four independent assays, we quantified the proportions of cells undergoing apoptosis and/or necrosis in the lower subfigures. * and ** denote *p* value < 0.05 and *p* value < 0.01, respectively.

Among the unions of 3700 genes, 406 were downregulated by both miR-140-3p and miR-126-5p transfection (Supplementary Table 2). To investigate the possible pathways, we conducted pathway enrichment analysis using Partek. As a result, 30 significantly enriched pathways were identified (Supplementary Table 3), and we were especially interested in the apoptosis pathway, since it offered the best explanation for the observations. To examine this pathway, we harvested neuronal cells transfected with scrambled control, miR-140-3p, or miR-126-5p for 24 h and then performed flow cytometry assays. As shown in Fig. 4, one flow assay demonstrated that overexpression of miR-126-5p and miR-140-3p reduced the number of cells driven into the late apoptosis stage (Q2 quadrant). Through four independent assays, miR-126-5p and miR-140-3p significantly lowered the proportions of cells undergoing late apoptosis. In addition to apoptosis, repressed necrosis activity (Q1 quadrant) was also observed in the cells transfected with miR-126-5p.

## Discussion

To the best of our knowledge, this study is the first to simultaneously present a miRNA biomarker panel for ADHD, the relationship between miRNAs and ADHD treatment response, and the molecular mechanism of the underlying association. We provide evidence that our previously established combinations of differential expression levels of miRNAs in WBCs are sufficient to differentiate the clinical samples of ADHD patients from those of healthy control subjects. Several miRNAs (miR-140-3p, miR-27a-3p, miR-486-5p, and miR-151-5p) potentially serve as biomarkers of the remission state of ADHD during a 1-year period of treatment with MPH. Furthermore, our in vitro study revealed that miR-140-3p and miR-126-5p facilitated the differentiation of HCN-2 human neuronal cells.

ADHD is a highly heritable neurodevelopmental disorder [45]. The pathophysiology of ADHD is associated with abnormalities in cortical development and network connectivity among several brain regions [46]. Our previous study using structural magnetic resonance imaging (MRI) indicated that the gray matter (GM) volume of the cingulate gyrus and left fusiform gyrus was positively correlated with the expression of miR-126-5p, miR-140-3p, and miR-30e-5p. [34] miRNAs participate in modulating gene expression and may regulate the cell proliferation and differentiation and synaptic plasticity of the CNS [47, 48]. Notably, circulating miRNA levels serve as noninvasive and sensitive biomarkers for reflecting the interactions between external stimuli and pathological neurodevelopment [49] and may be linked to ADHD manifestations [50]. One of the advantages of our study is that we used a global screening technique (NGS) to identify candidate miRNAs; global screening gave us the best chance at detecting novel miRNAs involved in the pathogenesis of ADHD [51]. Moreover, all previous studies that investigated similar topics lacked a validation group to confirm their findings. Consistent with our previous study, our results support the conclusion that the ADHD biomarker panel is reliable and feasible for clinical sample differentiation.

In our ADHD cohort, 54.3% of subjects met the criteria for remission after 12 months of follow-up, while 45.7% were not in remission. The findings of the current study indicated that four miRNAs (miR-140-3p, miR-27a-3p, miR-486-5p, and miR-151-5p) showed differential trends between responders and nonresponders during the 12-month follow-up. Among the responders, the improved ADHD symptoms during the 12-month follow-up period were positively correlated with increased expression of miR-140-3p, miR-27a-3p, miR-486-5p, and miR-151-5p. This finding indicates that these four miRNAs can potentially serve as biomarkers of remission during a one-year period of treatment with MPH. One study [22] reported that miRNA let-7d may serve as a potential diagnostic and therapeutic biomarker for children with ADHD. Another study [23] reported that the relative expression of miRNA-4655-3p and miRNA-7641 increased along with MPH or atomoxetine (ATX) treatment at 6 months. An animal study revealed that the rats exhibited impaired attention, worsened learning and memory abilities after they were administered the lentiviral vector containing Homer 1a-specific miRNA (Homer 1a-RNAi-LV) by intracerebroventricular injection [25]. These ADHD-like behaviors were improved by treatment of MPH. Pharmacogenetic studies illustrated individual variability in response to ADHD medications and provide clues for genetic predictors of response [52]. Response to medication may be affected by the expression of these pharmacogenomic genes, which are regulated by these miRNAs [53]. Although the mechanism and causality underlying the relationship between miRNAs and MPH treatment response remain unclear, we propose that the increased expression of miR-140-3p, miR-27a-3p, miR-486-5p, and miR-151-5p might regulate the expression levels of genes linked to the response to treatment of ADHD medication, or genes associated with underlying pathomechanism of ADHD. Future investigation regarding such association is warranted.

Among the three miRNAs, miR-30e-5p did not enhance HCN-2 differentiation, but miR-126-5p and miR-140-3p did. In addition, miR-140-3p was more effective than miR-126-5p (Fig. 3e, p-value < 0.05 for all indices). We were curious whether such a difference could be dosage-dependent, that is, if it resulted from unequal overexpression. In other words, the fact that miR-140-3p was the most effective and miR-30e-5p was ineffective was merely because miR-140-3p and miR-30e-5p had the highest and the lowest overexpression efficiencies, respectively. For this concern, we re-examined Supplementary Fig. 1 and found that miR-140-3p and miR-30e-5p individually had the lowest and middle overexpression efficiencies, respectively. Therefore, the ability of miR-140-3p and miR-126-5p to promote HCN-2 differentiation is an innate characteristic rather than a result of biased experimental manipulation. Therefore, our in vitro study provides evidence that the upregulation of miR-126-5p and miR-140-3p may facilitate the growth of the HCN-2 human neuronal cell line.

Our microarray and flow cytometry assays demonstrated that miR-140-3p or miR-126-5p treatment for 24 h significantly lowered the proportions of cells undergoing late apoptosis and/or necrosis activities. To the best of our knowledge, this is the first study to report the possible mechanism underlying the association between miR-140-3p/miR-126-5p and the pathophysiology of ADHD. Apoptosis refers to an active, programmed process of autonomous cellular dismantling, and necrosis is described as unplanned cell death resulting from environmental perturbations in the inflammatory process [54]. A previous in vitro study indicated that miR-126-5p may regulate H9c2 cell viability and apoptosis by targeting IL-17A under hypoxic conditions [55]. Another study revealed that overexpressing miR-140-3p may exert cytoprotective effects by alleviating inflammation and oxidative stress and reducing cell apoptosis in OGD/R [56]. Taken together with our findings, we suggest that miR-126-5p and miR-140-3p may facilitate neuronal growth and ameliorate apoptosis. The aforementioned miRNAs may be linked to susceptibility to ADHD and serve as ADHD biomarkers.

This study has several methodological issues and limitations that need to be addressed. First, miRNAs in WBCs were used as circulating biomarkers in this study, but miRNAs may also be present in exosomes within various bodily fluids [57]. As such, miRNA expression in peripheral blood may not illustrate the complete picture of epigenetic processes (i.e., the brain) related to ADHD. Although miRNA expression in circulating peripheral tissues has been reported to be correlated with that in neuronal tissues [58], whether these miRNAs affect brain function in the critical period of neurodevelopment needs to be further investigated. Second, since there was no data on miRNAs among patients without medication treatment or healthy subjects for comparison in this study, it remains uncertain whether the changes in miRNA levels were attributable to the effect of MPH treatment or natural maturation. Third, HCN-2 cell is a commonly used cerebral cortical cell line that stains positive for neuronal markers [59]. However, HCN-2 has scarcely been applied for an in vitro study on ADHD pathophysiology. Fourth, during the one-year period, environmental factors (e.g., diet, lifestyle, or other medications) might be confounding factors for the change in miRNAs. However, such factors were not measured in this study. Finally, the participants in this study were recruited from a single site in Taiwan. Further research should confirm whether the findings regarding the biomarker panel could be generalized to various ethnicities or countries.

In summary, this study is at the forefront of simultaneously illustrating a miRNA biomarker for ADHD and the molecular mechanism of the underlying association. We provide evidence that our previously established combination of differential expression levels of miRNAs in WBCs can be used to distinguish the clinical samples of ADHD patients from those of healthy control subjects. We also demonstrated that MPH may alter the expression of certain miRNAs, and miR-140-3p, miR-27a-3p, miR-486-5p, and miR-151-5p potentially serve as therapeutic markers of ADHD. Furthermore, our in vitro study revealed that miR-140-3p and miR-126-5p facilitate the growth of the HCN-2 human neuronal cell line. Our results demonstrate that the expression levels of miRNAs serve as ADHD biomarkers and are involved in the neurodevelopment/neuroprotection mechanisms that underlie ADHD pathophysiology.

## Supplementary information

Supplementary Table 1

Supplementary Table 2

Supplementary Table 3

Supplementary Fig. 1

Supplementary Fig. 2

Supplementary Fig. 3

## Data Availability

The data are available within the paper from the manuscript corresponding author on reasonable request.
