# Supplementary material for: MicroRNAs serve as prediction and treatment-response biomarkers of attention-deficit/hyperactivity disorder and promote the differentiation of neuronal cells by repressing the apoptosis pathway
Source: Transl Psychiatry. 2022 Feb 19;12:67. doi: 10.1038/s41398-022-01832-1 (PMC8858317; doi:10.1038/s41398-022-01832-1)
Supplement: Supplementary file 1 — Supplementary Table 1 [file 41398_2022_1832_MOESM1_ESM.doc]

**Supplementary Table 1. Baseline characteristics of patients with ADHD who were remitted (responders) and non-remitted (non-responders) after one-year methylphenidate treatment**

| **Characteristics** | **Responders**  **(N = 50)** | **Non-responders**  **(N = 42)** | **Statistic** | ***p*-value** |
| --- | --- | --- | --- | --- |
| **Sex** |  |  | 0.004 | 0.947 |
| Male | 39 (78) | 33 (78.6) |  |  |
| Female | 11 (22) | 9 (21.4) |  |  |
| **Age** (years) | 8.2 ± 1.8 | 9.3 ± 2.2 | -2.725 | 0.008* |
| **Height (cm)** | 130.7 ± 11.6 | 134.1 ± 14.1 | -1.223 | 0.225 |
| **Weight (kg)** | 31.1 ± 12.3 | 33.9 ± 13.1 | -1.058 | 0.293 |
| **WISC-IV** |  |  |  |  |
| FSIQ | 99.2 ± 11.1 | 99.3 ± 9.2 | -0.021 | 0.983 |
| Verbal Comprehension Index | 103.6 ± 12.1 | 101.2 ± 9.2 | 1.089 | 0.279 |
| Perceptual Reasoning Index | 99.8 ± 13.9 | 99.6 ± 11.6 | 0.076 | 0.939 |
| Working Memory Index | 101.0 ± 13.1 | 100.8 ± 11.6 | 0.073 | 0.942 |
| Processing Speed Index | 92.9 ± 9.1 | 95.7 ± 10.3 | -1.401 | 0.165 |
| **Clinical measures** |  |  |  |  |
| ADHD-RS (I) | 22.8 ± 4.6 | 23.1 ± 4.4 | -0.321 | 0.749 |
| ADHD-RS (H) | 23.6 ± 4.9 | 23.2 ± 5.0 | 0.417 | 0.678 |
| **Methylphenidate dose** (mg) | 27.0 ± 8.0 | 26.9 ± 7.9 | 0.082 | 0.935 |

^a^ Data are expressed as N (%) or Mean ± SD; FSIQ, Full Scale Intelligence Quotient; H, hyperactivity/impulsivity scores; I, inattention scores; WISC-IV, Wechsler Intelligence Scale for Children–Fourth Edition; **p*<0.05
