# Supplementary material for: MicroRNAs serve as prediction and treatment-response biomarkers of attention-deficit/hyperactivity disorder and promote the differentiation of neuronal cells by repressing the apoptosis pathway
Source: Transl Psychiatry. 2022 Feb 19;12:67. doi: 10.1038/s41398-022-01832-1 (PMC8858317; doi:10.1038/s41398-022-01832-1)
Supplement: Supplementary file 2 — Supplementary Table 2 [file 41398_2022_1832_MOESM2_ESM.doc]

**Supplementary Table 2. The list of the 406 genes simultaneously down-regulated with miR-140-3p or miR-126-5p mimic transfection.**

| **Gene Symbol** |
| --- |
| ISG15 |
| AGRN |
| FBXO6 |
| EPB41 |
| AZIN2 |
| IFI44L |
| IFI44 |
| RP4-641G12.3 |
| GBP6 |
| GBP1P1 |
| VCAM1 |
| MOV10 |
| HIST2H4B |
| RP11-98D18.15 |
| IFI16 |
| BLZF1 |
| RP5-1114G22.1 |
| PLA2G4A |
| RP4-608O15.4 |
| AGMAT |
| RP4-781L3.1 |
| IFI6 |
| SNORA44 |
| RNF19B |
| FUBP1 |
| MCOLN3 |
| GBP3 |
| GBP1 |
| GBP4 |
| SLC16A4 |
| TXNIP |
| CTSS |
| LYSMD1 |
| RFX5 |
| ADAR |
| LOC100506023 |
| ANGPTL1 |
| RP11-480I12.10 |
| LINC01139 |
| NPPA-AS1_2 |
| RP11-337C18.8 |
| GBP2 |
| SLC1A4 |
| AC007422.1 |
| AC012493.1 |
| RNU6-436P |
| LOC101927027 |
| LOC101927482 |
| SPATS2L |
| CASP10 |
| CASP8 |
| SNORD70 |
| SNORD51 |
| LOC101928103 |
| SP140L |
| SP100 |
| RN7SL834P |
| CMPK2 |
| EIF2AK2 |
| PNPT1 |
| TACR1 |
| NMI |
| IFIH1 |
| RP11-512G4.1 |
| STAT1 |
| AC067945.3 |
| CLK1 |
| IKZF2 |
| SP110 |
| RSAD2 |
| FAM228B |
| CNOT10 |
| MYD88 |
| ZNF502 |
| CCRL2 |
| UBA7 |
| STAB1 |
| DTX3L |
| PARP14 |
| ACKR4 |
| RASA2 |
| YEATS2 |
| SNORD66 |
| RTP4 |
| GRIP2 |
| RP11-640L9.2 |
| TRANK1 |
| AC011816.1 |
| UBA7 |
| PARP9 |
| PLSCR1 |
| RP11-78O22.1 |
| TNFSF10 |
| GNB4 |
| ABCC5 |
| MB21D2 |
| PLSCR2 |
| LAP3 |
| ARL9 |
| THEGL |
| THAP6 |
| HERC6 |
| HERC5 |
| RP11-254A24.2 |
| CCDC109B |
| C4orf33 |
| IL15 |
| GUCY1B3 |
| TLR3 |
| RP11-478C6.2 |
| LDB2 |
| GABRA4 |
| AC092597.3 |
| BTC |
| CXCL9 |
| CXCL10 |
| CXCL11 |
| SCARB2 |
| PPM1K |
| HERC6 |
| DDX60 |
| ARAP2 |
| TLR1 |
| RASGEF1B |
| SCLT1 |
| DDX60L |
| CTD-2194D22.3 |
| TMEM171 |
| ERAP2 |
| C5orf56 |
| C5orf56 |
| VTRNA1-2 |
| RP11-124N3.2 |
| RICTOR |
| SLC38A9 |
| MTX3 |
| MCTP1 |
| RN7SL689P |
| IRF1 |
| SNORD63 |
| SNORD63 |
| CTB-79E8.2 |
| RAB24 |
| DSP |
| GMPR |
| TRIM38 |
| BTN3A2 |
| BTN3A3 |
| BTN2A1 |
| HLA-H |
| HLA-E |
| MSH5 |
| RP1-50J22.4 |
| LAP3P2 |
| CMTR1 |
| NCOA7 |
| RP11-439L18.1 |
| RP1-95L4.3 |
| RP11-532F6.3 |
| RP4-625H18.2 |
| ZSCAN23 |
| PSMB8 |
| TAP1 |
| RP3-391O22.3 |
| RP1-179N16.3 |
| ETV7 |
| EPHA7 |
| SLC2A12 |
| U8 |
| CFB |
| PSMB8-AS1 |
| PSMB9 |
| TAP2 |
| FAM46A |
| BUD31 |
| TMEM140 |
| RP11-134L10.1 |
| NUP205 |
| FTSJ2 |
| DGKB |
| ANKMY2 |
| IGF2BP3 |
| AMPH |
| DDC |
| ERV3-1 |
| GTF2IP7 |
| SAMD9 |
| SAMD9L |
| MIR591 |
| PTN |
| ZC3HAV1 |
| PARP12 |
| LOC100506302 |
| ZNF107 |
| SLC25A37 |
| LY6E |
| CD274 |
| PDCD1LG2 |
| CHMP5 |
| GLIPR2 |
| PSAT1 |
| IDNK |
| TDRD7 |
| STX17 |
| TRAF2 |
| DDX58 |
| KIF27 |
| TRIM14 |
| S100G |
| FAM122C |
| RP13-150K15.1 |
| CXorf23 |
| RLIM |
| HDX |
| ACTRT1 |
| FAM122B |
| MASTL |
| CFL1P1 |
| IFIT2 |
| IFIT3 |
| IFIT1 |
| IFIT5 |
| PCGF5 |
| RBM20 |
| ACSL5 |
| CPEB3 |
| SLC25A28 |
| LIPA |
| PRRG4 |
| SERPING1 |
| OR5AN1 |
| RARRES3 |
| LOC105369364 |
| UNC93B6 |
| IL18BP |
| IRF7 |
| TRIM21 |
| TRIM5 |
| TRIM22 |
| RP11-732A19.1 |
| NRIP3 |
| AGBL2 |
| UBE2L6 |
| PRPF19 |
| SLC15A3 |
| CDC42BPG |
| BATF2 |
| UNC93B1 |
| IFITM9P |
| RP11-685N10.1 |
| KBTBD3 |
| BLID |
| IFITM1 |
| TRIM34 |
| TRIM6-TRIM34 |
| TRIM22 |
| OR56B1 |
| TEX12 |
| RBM7 |
| CASP1 |
| CARD16 |
| FGFR1OP2 |
| OAS3 |
| OAS2 |
| P2RX7 |
| KLRAP1 |
| STAT2 |
| DDIT3 |
| ALDH1L2 |
| TMEM116 |
| OASL |
| LOC101927415 |
| RP11-749H20.1 |
| C1S |
| OAS1 |
| C1R |
| SPATA13 |
| RP11-756A22.7 |
| PSME2P2 |
| PHF11 |
| MIR4703 |
| LINC01075 |
| GPC6 |
| IPPKP1 |
| PARP4 |
| EPSTI1 |
| SNRPGP11 |
| SLITRK6 |
| DOCK9 |
| UCHL3 |
| TNFSF13B |
| RP11-298I3.4 |
| PSME1 |
| REC8 |
| GSTZ1 |
| IFI27 |
| RP11-638I2.10 |
| ZFYVE21 |
| RP11-247L20.4 |
| CDKL1 |
| WARS |
| IRF9 |
| PSME2 |
| SNORD109B |
| FAM98B |
| LIPC |
| GDPGP1 |
| SPG11 |
| RP11-507J18.5 |
| CTD-2323K18.2 |
| TMEM62 |
| TRIM69 |
| PARP16 |
| FLJ42627 |
| ABCC1 |
| NLRC5 |
| PPL |
| MLKL |
| ASPA |
| XAF1 |
| CCL2 |
| CCL8 |
| TTC25 |
| CNP |
| IFI35 |
| LRRC37A2 |
| RP11-670E13.5 |
| RAB37 |
| ICT1 |
| RNF213 |
| ANKFY1 |
| CXCL16 |
| RP11-609D21.3 |
| RP11-354P11.8 |
| RP11-227G15.6 |
| KRT19 |
| DHX58 |
| TRIM25 |
| 10-Mar |
| FTSJ3 |
| CD68 |
| CCDC144A |
| LRRC37B |
| SMCHD1 |
| RP11-513M1.1 |
| ZCCHC2 |
| RP11-120K19.3 |
| CNDP2 |
| PTPN2 |
| ZNF396 |
| RIT2 |
| CYB5A |
| ATP8B1 |
| C19orf66 |
| MVB12A |
| MAST3 |
| ZNF101 |
| ZNF431 |
| CATSPERG |
| USP29 |
| BST2 |
| IL12RB1 |
| LOC101929144 |
| C19orf12 |
| LGI4 |
| ZFP14 |
| PRKD2 |
| CTC-471J1.2 |
| VN1R4 |
| MFSD12 |
| ZNF564 |
| RBCK1 |
| REM1 |
| RNF114 |
| OGFR |
| NRSN2-AS1 |
| SAMHD1 |
| HELZ2 |
| ZNFX1 |
| CYYR1-AS1 |
| MX2 |
| MX1 |
| ADARB1 |
| C21orf91 |
| USP18 |
| CTA-390C10.10 |
| DEPDC5 |
| APOL6 |
| APOL1 |
| RP3-508I15.19 |
| TTC38 |
| MIR3201 |
| CTA-256D12.11 |
| APOL3 |
| APOL2 |
| RP4-756G23.5 |
| APOBEC3G |
| TYMP |
| CHKB |
| CCDC84 |
| ZNF546 |
| ZNF35 |
| NDUFA6 |
| AL772161.2 |
| TRAPPC4 |
| SLC25A26 |
