# Supplementary material for: MicroRNAs serve as prediction and treatment-response biomarkers of attention-deficit/hyperactivity disorder and promote the differentiation of neuronal cells by repressing the apoptosis pathway
Source: Transl Psychiatry. 2022 Feb 19;12:67. doi: 10.1038/s41398-022-01832-1 (PMC8858317; doi:10.1038/s41398-022-01832-1)
Supplement: Supplementary file 3 — Supplementary Table 3 [file 41398_2022_1832_MOESM3_ESM.doc]

**Supplementary Table 3.** The significant pathways enriched by 406 genes simultaneously down-regulated with miR-140-3p or miR-126-5p mimic transfection. The enrichment p-value was calculated based on hyper-geometric distribution.

| **Pathway Name** | **Enrichment Score** | **Enrichment p-value** | **Pathway ID** |
| --- | --- | --- | --- |
| Influenza A | 27.0377 | 1.81E-12 | kegg_pathway_169 |
| NOD-like receptor signaling pathway | 25.7730 | 6.41E-12 | kegg_pathway_75 |
| Hepatitis C | 17.7721 | 1.91E-08 | kegg_pathway_166 |
| Epstein-Barr virus infection | 15.9193 | 1.22E-07 | kegg_pathway_172 |
| Measles | 15.4555 | 1.94E-07 | kegg_pathway_168 |
| RIG-I-like receptor signaling pathway | 14.5333 | 4.88E-07 | kegg_pathway_76 |
| Herpes simplex virus 1 infection | 14.1621 | 7.07E-07 | kegg_pathway_171 |
| Necroptosis | 13.4829 | 1.39E-06 | kegg_pathway_226 |
| Toll-like receptor signaling pathway | 9.0020 | 1.23E-04 | kegg_pathway_74 |
| TNF signaling pathway | 8.6443 | 1.76E-04 | kegg_pathway_85 |
| Antigen processing and presentation | 8.1084 | 3.01E-04 | kegg_pathway_72 |
| Human immunodeficiency virus 1 infection | 6.4348 | 1.60E-03 | kegg_pathway_239 |
| Kaposi sarcoma-associated herpesvirus infection | 6.1400 | 2.15E-03 | kegg_pathway_227 |
| Pertussis | 5.8811 | 2.79E-03 | kegg_pathway_156 |
| Hepatitis B | 5.7719 | 3.11E-03 | kegg_pathway_167 |
| Cytosolic DNA-sensing pathway | 5.1504 | 5.80E-03 | kegg_pathway_77 |
| Chemokine signaling pathway | 4.8163 | 8.10E-03 | kegg_pathway_28 |
| ABC transporters | 4.7070 | 9.03E-03 | kegg_pathway_1 |
| Proteasome | 4.6301 | 9.75E-03 | kegg_pathway_11 |
| NF-kappa B signaling pathway | 4.5617 | 1.04E-02 | kegg_pathway_29 |
| Viral carcinogenesis | 4.4678 | 1.15E-02 | kegg_pathway_175 |
| C-type lectin receptor signaling pathway | 4.4260 | 1.20E-02 | kegg_pathway_236 |
| Apoptosis | 4.3940 | 1.24E-02 | kegg_pathway_50 |
| Arginine and proline metabolism | 4.3418 | 1.30E-02 | kegg_pathway_266 |
| Legionellosis | 4.0191 | 1.80E-02 | kegg_pathway_157 |
| Cytokine-cytokine receptor interaction | 3.9386 | 1.95E-02 | kegg_pathway_27 |
| African trypanosomiasis | 3.5130 | 2.98E-02 | kegg_pathway_160 |
| Primary immunodeficiency | 3.5130 | 2.98E-02 | kegg_pathway_202 |
| Human papillomavirus infection | 3.2066 | 4.05E-02 | kegg_pathway_229 |
| Tuberculosis | 3.0996 | 4.51E-02 | kegg_pathway_165 |
