# Supplementary material for: MicroRNAs serve as prediction and treatment-response biomarkers of attention-deficit/hyperactivity disorder and promote the differentiation of neuronal cells by repressing the apoptosis pathway
Source: Transl Psychiatry. 2022 Feb 19;12:67. doi: 10.1038/s41398-022-01832-1 (PMC8858317; doi:10.1038/s41398-022-01832-1)
Supplement: Supplementary file 4 — Supplementary Fig. 1 [file 41398_2022_1832_MOESM4_ESM.pdf]

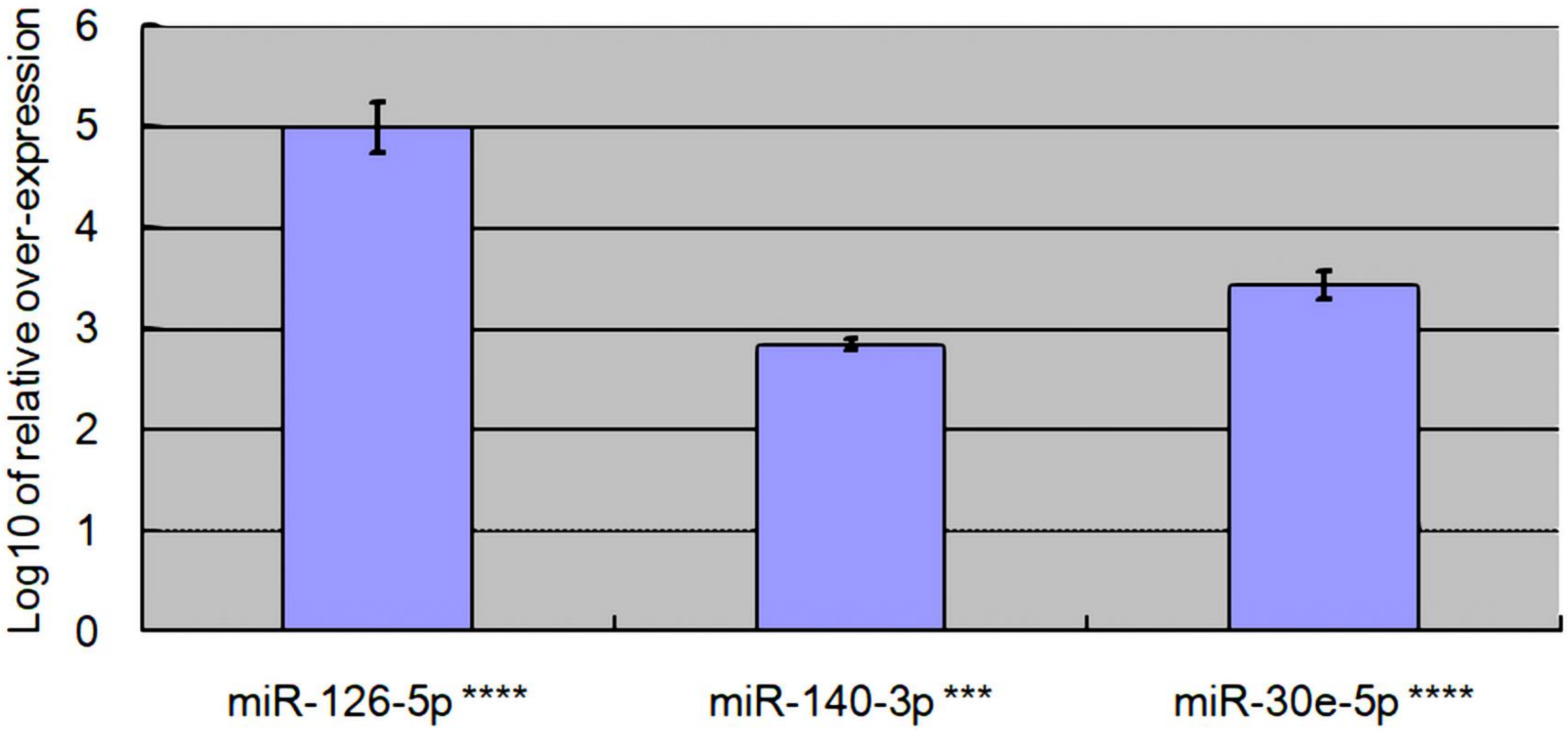

Supplementary Fig. 1. Enhanced expression levels of miRNAs with mimic transfection. We enhanced miRNA expression levels by mimic transfection in HCN-2 cells. HCN-2 cells transfected for 24 h were harvested from qPCR assays.
