# Supplementary material for: MicroRNAs serve as prediction and treatment-response biomarkers of attention-deficit/hyperactivity disorder and promote the differentiation of neuronal cells by repressing the apoptosis pathway
Source: Transl Psychiatry. 2022 Feb 19;12:67. doi: 10.1038/s41398-022-01832-1 (PMC8858317; doi:10.1038/s41398-022-01832-1)
Supplement: Supplementary file 5 — Supplementary Fig. 2 [file 41398_2022_1832_MOESM5_ESM.pdf]

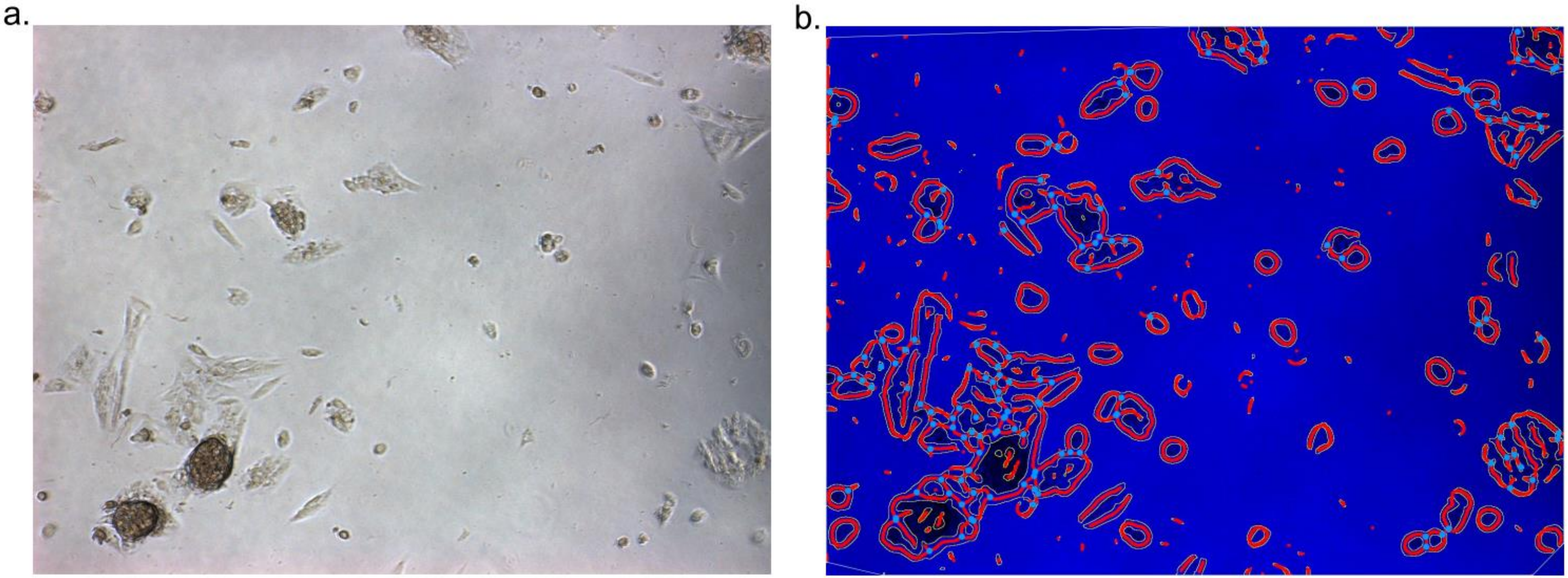

Supplementary Fig. 2. Illustration of the use of AngioTool to analyze pictures recording the HCN-2 differentiation pattern. (a) The differentiation pattern of HCN-2 cells recorded with a camera. (b) Since neuronal cells tend to differentiate into network (tube, also called vessel) structures, we used AngioTool to analyze their differentiation patterns. The thin yellow lines sketch the outline of vessels, used for recording the area of vessels. The thick red lines record the lengths of vessels. The blue dots represent junctions of vessel structures.
