# Supplementary material for: MicroRNAs serve as prediction and treatment-response biomarkers of attention-deficit/hyperactivity disorder and promote the differentiation of neuronal cells by repressing the apoptosis pathway
Source: Transl Psychiatry. 2022 Feb 19;12:67. doi: 10.1038/s41398-022-01832-1 (PMC8858317; doi:10.1038/s41398-022-01832-1)
Supplement: Supplementary file 6 — Supplementary Fig. 3 [file 41398_2022_1832_MOESM6_ESM.pdf]

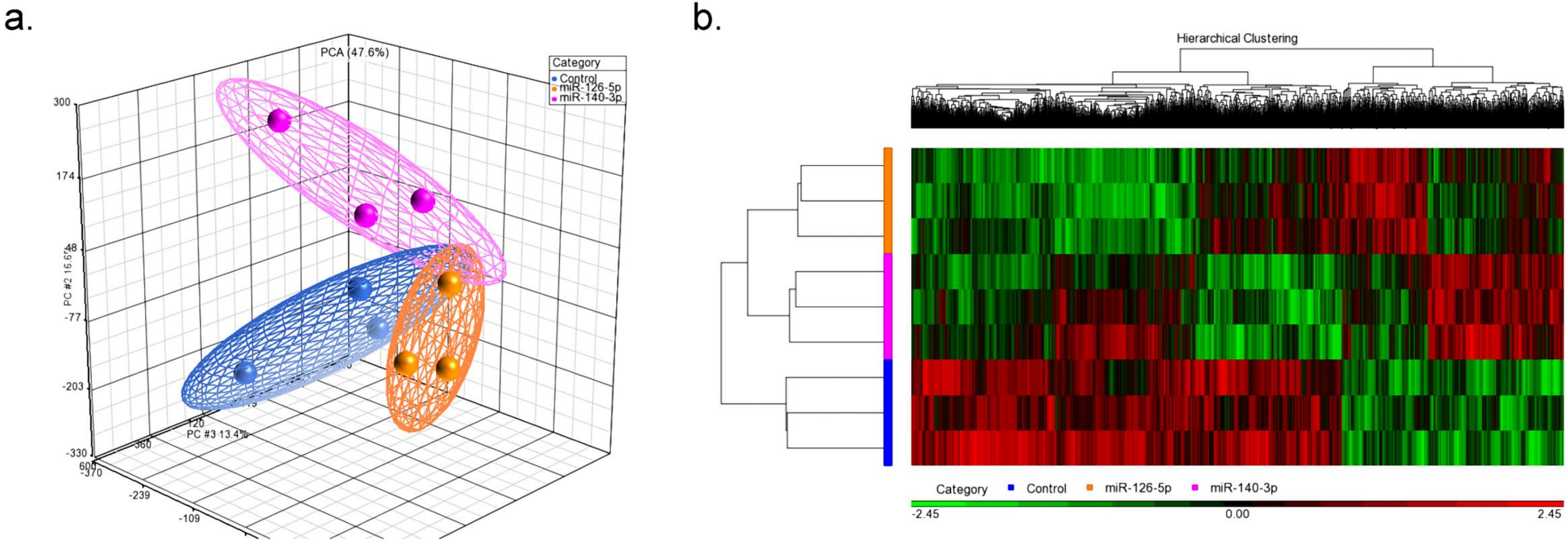

Supplementary Fig. 3. The overall results of microarray assays on HCN-2 cells with different miRNA mimics. We used microarray assays to examine the gene expression profiles of HCN-2 cells transfected with miRNA mimic. (a) The PCA plot demonstrated that the three sets had specific and discriminative gene expression profiles. (b) Although with their own specific profiles, HCN-2 cells transfected with miR-126-5p and miR-140-3p had closer and similar gene expression profiles when clustered with the union of differentially expressed genes.
